# ASSESSING HOW AGE, GENDER, RACE, AND EDUCATION AFFECT THE RELATIONSHIPS BETWEEN COGNITIVE DOMAINS AND OLFACTION

**DOI:** 10.1093/geroni/igad104.0235

**Published:** 2023-12-21

**Authors:** Selena Zhong, Kristen Wroblewski, Edward Laumann, Martha McClintock, Jayant Pinto

**Affiliations:** NORC, Chicago, Illinois, United States; University of Chicago, Chicago, Illinois, United States; University of Chicago, Chicago, Illinois, United States; University of Chicago, Chicago, Illinois, United States; University of Chicago, Chicago, Illinois, United States

## Abstract

The associations between cognitive domains and odor identification are well-established, but how sociodemographic variables affect these relationships is less clear; using the survey-adapted Montreal Cognitive Assessment instrument (MoCA-SA), we assess how age, gender, race, and education shape these relationships. First, we used two different methods, cluster analysis and multidimensional scaling, to empirically derive distinct cognitive domains from the MoCA-SA since it is unclear whether the MoCA-SA can be disaggregated into cognitive domains. We then used ordinal logistic regression to test whether these empirically derived cognitive domains were associated with odor identification and how sociodemographic variables modified these relationships. We identified five out of the six theoretical cognitive domains, with the language domain unable to be identified. We found that odor identification was associated with episodic memory, visuospatial ability, and executive function. Stratified analyses by sociodemographic variables reveal that the associations between some of the cognitive domains and odor identification varied by age, gender, or race, but not by education. These results suggest that 1) the MoCA-SA can be used to identify cognitive domains in survey research and 2) the performance of smell tests as a screener for cognitive decline may potentially be weaker in certain subpopulations.

